# Extrafloral Nectaries in Woody Plants: Toward an Integrated Framework for Ant-Mediated Plant Defense

**DOI:** 10.3390/plants15132100

**Published:** 2026-07-07

**Authors:** Fangming Liu, Qifei Cai, Victor Busov, Zhen Liu

**Affiliations:** 1 Henan Province Engineering Technology Research Center for *Idesia*, Zhengzhou 450046, China; fangmingliu-0930@henau.edu.cn (F.L.); cai_qifei@henau.edu.cn (Q.C.); 2 National Forestry and Grassland Administration Key Laboratory for Central Plains Forest Resources Cultivation, Zhengzhou 450046, China; 3 College of Forestry, Henan Agricultural University, Zhengzhou 450046, China; 4 College of Forest Resources and Environmental Science, Michigan Technological University, Houghton, MI 49931, USA

**Keywords:** extrafloral nectaries, ant-mediated plant defense, woody plant, nectar chemistry, *Idesia polycarpa*, multi-trophic interactions

## Abstract

Extrafloral nectaries (EFNs)—secretory structures that recruit predatory arthropods as indirect defenders against herbivory—have been studied almost exclusively in herbaceous systems, leaving a critical gap in our understanding of plant defense in woody plants. In trees, secondary growth, ontogenetic regulation, and seasonal phenology generate structural and temporal heterogeneity in EFN expression poorly captured by existing frameworks. We synthesize evidence across structural, chemical, and functional dimensions of EFN biology to develop an integrated framework for perennial trees. Our analysis suggests that methodological limitations in EFN detection likely bias the current chemical record of woody EFNs, while incomplete chemical characterization constrains the evaluation of their ecological functions. Existing evidence confirms that herbivory-inducible EFN secretion and ant-mediated defense operate in trees, but their generality across ontogenetic stages, seasons, and woody lineages remains unresolved. We propose *Idesia polycarpa* (Salicaceae) as a candidate model system for mechanistic EFN research in trees, supported by confirmed secretory activity, documented ant visitation, and a well-characterized genomic and phylogenetic context, and offer four testable predictions to guide future work on indirect defense in long-lived woody plants.

## 1. Introduction

Nectaries are specialized secretory structures that produce nutrient-rich nectar, mediating fundamental ecological interactions between plants and animals [1]. In angiosperms, they are broadly categorized into floral nectaries (FNs) and extrafloral nectaries (EFNs): FNs primarily facilitate pollination, while EFNs recruit mutualistic arthropods—particularly ants—as indirect defenders against herbivory [1,2]. EFNs are evolutionarily labile traits that have arisen repeatedly across more than 100 angiosperm families, reflecting strong ecological selection rather than deep homology [3,4], and their defensive function—attracting predatory arthropods that reduce herbivore damage and enhance plant fitness—is well-supported across diverse plant–ant systems [2,5]. This empirical understanding derives predominantly from herbaceous or short-lived systems, where EFNs are conspicuous and experimentally tractable, leaving EFN biology in long-lived woody plants comparatively underexplored.

In woody plants—here defined as trees, shrubs, and woody lianas sharing the common property of stems reinforced by secondary xylem—EFNs have been known since at least the late nineteenth century [6] but remain comparatively poorly characterized relative to herbaceous systems. Although this broad category encompasses diverse growth forms, the present review focuses primarily on trees: the available empirical literature on woody EFNs, sparse as it is, derives overwhelmingly from herbaceous-system comparisons and isolated tree case studies rather than from systematic arboreal sampling, and it is in trees that the challenges posed by secondary growth, extended ontogeny, and architectural complexity are most pronounced. These perennial plants frequently possess cryptic, seasonally variable, or developmentally dynamic EFNs that are difficult to detect and study, leading to their historical underrepresentation in anatomical, chemical, and ecological research [3,7]. Secondary growth, complex canopy architecture, and pronounced ontogenetic shifts create spatial and temporal heterogeneity in nectar production that existing models of EFN function rarely capture [8,9,10]. Consequently, critical questions persist: How do EFN morphology, placement, and chemistry vary across tree organs? To what extent is EFN secretion inducible in trees? And how does this variation shape ant recruitment, herbivore suppression, and community-level interactions in forest ecosystems?

Addressing these questions requires integrating knowledge across scales—from nectary structure and nectar chemistry to ant behavior and ecosystem dynamics. Yet the biology of EFN-bearing trees remains insufficiently characterized to translate these possibilities into practice: the mechanistic links between EFN structure, nectar chemistry, and defensive outcome in perennial woody systems are largely unresolved, and existing frameworks have not addressed these dimensions in combination for woody plants—a gap the present synthesis aims to fill.

This review addresses these questions through an integrated framework organized across three interconnected dimensions—structural, chemical, and functional—grounded in a comparative analysis of FNs and EFNs (Section 2) that supports the conceptual foundation neither pollination biology nor herbaceous EFN models alone can supply. Building on this foundation, the review examines the structural diversity and cryptic morphology of EFNs in woody plants (Section 3), the chemical ecology of extrafloral nectar (Section 4), and the evidence for EFN-mediated indirect defense from the individual to the ecosystem scale (Section 5), culminating in the proposal of *Idesia polycarpa* as a well-supported candidate model system—with confirmed secretory activity and documented ant visitation—positioned to anchor an integrated research program (Section 6).

## 2. Floral and Extrafloral Nectaries: A Comparative Framework

Nectaries are secretory structures that produce nectar as a medium of exchange between plants and animals, but floral nectaries (FNs) and extrafloral nectaries (EFNs) have evolved to serve structurally distinct ecological purposes. FNs are positioned within flowers and deliver nectar as a reward to animal pollinators, directly enhancing plant reproductive success [11,12,13]. EFNs, by contrast, are located on vegetative or reproductive structures outside the flower and recruit predatory arthropods—principally ants—as indirect defenders against herbivory [1,2]. Although both nectary types share the same underlying secretory machinery, they diverge markedly in developmental origin, chemical profile, regulatory logic, and ecological function. Establishing this comparative framework is a necessary foundation for understanding the distinctive biology of EFNs in woody plants.

### 2.1. Developmental Origins and Evolutionary Trajectories

The contrasting evolutionary histories of FNs and EFNs reflect their different selective regimes. FN development is governed by conserved transcriptional networks within the broader context of floral organogenesis, producing developmental canalization that constrains nectary architecture within lineages and synchronizes secretion with pollinator-mediated reproductive timing [14,15,16].

EFNs stand in sharp contrast. Defined by defensive function rather than conserved developmental origin, they have evolved independently across more than 100 angiosperm families with no requirement for organ homology [3,4]. Closely related species can differ dramatically in EFN presence, position, and structure, reflecting their status as evolutionarily labile traits readily gained or lost in response to ecological pressures—particularly the local availability of effective ant mutualists—rather than traits fixed by phylogenetic constraint [8]. EFN placement follows a strategic defensive logic: glands are consistently biased toward developmentally costly, herbivore-vulnerable tissues, directing ant patrols to the sites of highest defensive need [17,18]. For woody plants specifically, this evolutionary lability has a direct and consequential implication: EFNs may be present, absent, or structurally transformed within a single genus or even species complex, and their morphological diversity—from prominent petiolar glands to cryptic parenchymatous patches—cannot be predicted from phylogenetic position alone.

### 2.2. Nectar Chemistry: Divergent Profiles for Divergent Functions

FNs and EFNs produce chemically distinct nectars tuned to their contrasting ecological roles: floral nectar is matched to pollinator sensory and nutritional requirements [19,20], whereas extrafloral nectar is compositionally oriented toward sustaining resident ant colonies and filtering mutualist identity rather than attracting transient visitors [21]. The mechanistic basis of this distinction—sugar profiles, amino acid content, and secondary metabolite function—is examined in detail in Section 4. Here, the key point is that this chemical divergence carries direct implications for woody plant research: in forest ecosystems, where arthropod visitor diversity substantially exceeds that of herbaceous systems, the partner-filtering function of EFN chemistry is correspondingly more consequential yet remains largely uncharacterized across woody lineages [7,22].

### 2.3. Regulatory Logic: Constitutive Reward Versus Inducible Defense

The regulatory logic governing nectar secretion differs between the two nectary types—a difference with direct bearing on the study of EFN biology in perennial trees. FN secretion is constitutive and synchronized with the flowering period [23,24], a pattern that offers no template for understanding the dynamic, herbivore-responsive secretion that characterizes EFN function. EFN secretion, in well-studied herbaceous systems, is frequently inducible: nectar output can be upregulated rapidly by herbivore damage or jasmonate signaling, representing a cost-saving dynamic response that concentrates defensive investment when and where threat is perceived—a regulatory logic that contrasts fundamentally with the constitutive, developmentally canalized output of FNs [2,25,26]. Whether this inducible logic operates comparably in mature trees remains a critical and unresolved question—secondary growth, complex canopy architecture, and extended ontogeny may impose constraints on phenotypic plasticity that shift secretion toward more developmentally pre-programmed patterns [10,27]. The constitutive, pollinator-synchronized logic of FN biology offers no guidance here; understanding inducibility in perennial trees requires investigation on its own terms and remains the most pressing unresolved question in woody plant EFN biology.

### 2.4. Synthesis: The Comparative Framework as a Foundation for Woody Plant EFN Research

FNs and EFNs deploy a shared secretory mechanism toward contrasting ecological ends: pollination reward versus indirect defense, developmental canalization versus evolutionary lability, and constitutive output versus dynamic induction. These distinctions define not merely an academic typology but a set of biological constraints and expectations that directly frame the study of EFNs in these systems. Critically, neither the FN model nor the herbaceous EFN model can be straightforwardly extrapolated to trees. The pollinator-mediated logic of FN biology is irrelevant to EFN function, while the inducible, plastic model well-characterized in annual herbs may not translate to long-lived perennials, where structural complexity and extended ontogeny introduce spatiotemporal heterogeneity that existing frameworks incompletely capture [2,4,28].

## 3. Structural Diversity and Distribution of Extrafloral Nectaries

FN and EFN biology diverge in developmental origin, chemical profile, and regulatory logic—divergences that make the structural dimension the necessary starting point for understanding EFN biology in woody plants, because EFN morphology, anatomy, and phylogenetic distribution determine what can be observed in the field, what has been systematically overlooked, and why trees have remained so conspicuously underrepresented in EFN research relative to herbaceous systems. This section examines the morphological diversity, internal anatomy, organ-level distribution, and phylogenetic context of EFNs, then addresses the structural and methodological challenges that make these structures particularly difficult to detect and study in these perennial systems—challenges that set the stage for the chemical and functional analyses developed in Section 4 and Section 5.

### 3.1. Structural Diversity and Organ-Level Distribution

EFNs are defined by defensive function rather than by a conserved anatomical plan, and this functional definition has a direct structural consequence: EFN morphology is extraordinarily heterogeneous. Across angiosperms, EFNs span a continuous structural spectrum from morphologically prominent, elevated cup-shaped or discoid glands to anatomically inconspicuous flattened patches of secretory parenchyma that are barely distinguishable from surrounding tissue without histochemical examination [29,30]. This structural continuum is not incidental but reflects the absence of any developmental constraint on EFN form because EFNs have no conserved organ of origin and have evolved independently across more than 100 angiosperm families; there is no shared developmental template to canalize their morphology [3,4].

A critical principle follows directly from this morphological heterogeneity: structural conspicuousness does not predict ecological function. Secretory rate and nectar chemical composition—rather than gland size or structural prominence—are the primary determinants of ant recruitment intensity and mutualist fidelity. Anatomically inconspicuous EFNs can therefore sustain protective ant attendance comparable to that recorded at morphologically prominent glands [31]. This was demonstrated directly by González-Teuber et al. [32], who showed that increasing host investment in extrafloral nectar improved the efficiency of ant-mediated defense independent of gland morphology—with secretory output, not structural form, governing the quality of the defensive service received. This decoupling of form from function is not merely a biological curiosity; it has an immediate and general methodological consequence: surveys that rely on visual identification of gland-like structures will systematically underestimate EFN diversity, regardless of the plant system under investigation. Histochemical detection of secretory activity, rather than morphological inspection alone, is therefore a prerequisite for accurate documentation of EFN occurrence in woody plant systems.

The organ-level distribution of EFNs across the plant body is similarly broad but far from random. EFNs occur on nearly every aboveground organ type, including leaf blades, leaf margins, petioles, stipules, stems, rachises, and inflorescence axes [29,30,33], but this anatomical breadth is not randomly distributed: placement is consistently biased toward tissues that are both developmentally costly to produce and highly vulnerable to herbivory, including young expanding leaves, actively elongating shoot apices, developing reproductive structures, and the petioles of actively photosynthesizing leaves [17,18]. This spatial bias concentrates ant patrols precisely at the sites where the fitness cost of herbivory is highest, a pattern broadly consistent with the predictions of optimal defense theory [34]. That observed EFN placement aligns with these predictions—rather than reflecting developmental constraints on where secretory tissue can form—suggests that the spatial logic of EFN distribution is shaped at least in part by selection acting on defensive outcomes, though the degree to which optimal defense theory captures the full range of factors governing EFN placement across species remains an open question.

### 3.2. Internal Anatomy and Nectar Release

The external morphological diversity is underlain by a corresponding diversity in internal anatomical organization, which directly governs secretory capacity and nectar output. At the cellular level, EFNs are typically composed of one or more layers of secretory epithelium—densely cytoplasmic cells with prominent nuclei, abundant mitochondria, and well-developed endoplasmic reticulum—overlying a vascularized parenchymatous core that supplies the substrates for nectar synthesis [11,29]. The degree of vascularization, the thickness of the secretory epithelium, and the nature of the connection to the phloem or xylem supply vary substantially across EFN types and are correlated with secretory rate and nectar volume [7,33]. Nectar release occurs through one of three anatomical routes—nectarostomata, cuticular pores, or modified glandular trichomes—and this route, rather than being a neutral structural detail, directly shapes nectar viscosity, evaporation rate, and accessibility to visiting arthropods, with consequences for the composition and quality of the attendant ant community [11].

For trees and shrubs, almost nothing is known about EFN internal anatomy beyond descriptive accounts of a small number of species [7,35]. Given that secondary growth, modified phloem architecture, and pronounced ontogenetic shifts distinguish the vascular supply to EFNs in these perennial species from that in herbs, anatomical organization may differ in ways that are not predictable from herbaceous models. Systematic anatomical investigation—combining histochemical staining, transmission electron microscopy, and vascular tracing across organ types and developmental stages—is consequently essential for predicting how vascular architecture and ontogenetic context shape secretory capacity in perennial woody systems.

### 3.3. Phylogenetic Distribution and Evolutionary Lability

The structural and distributional diversity is the phenotypic expression of a deeper evolutionary property: EFNs are among the most evolutionarily labile traits known in angiosperms. Their repeated independent origins across distantly related lineages—documented in more than 100 families—reflect ecological opportunity rather than phylogenetic inheritance, with gains and losses driven primarily by the local availability of effective ant mutualists rather than by constraint from shared developmental pathways [3,4].

Several phylogenetic patterns illuminate this evolutionary history. The near-absence of EFNs in gymnosperms and early-diverging angiosperm lineages, followed by their proliferation in more derived clades, suggests that the ecological preconditions for EFN evolution—most importantly, the presence of diverse and ecologically dominant ant faunas—were not in place until the radiation and diversification of ants during the Cretaceous and Paleogene [3,36]. This temporal correspondence is consistent with a co-evolutionary interpretation of EFN origins, in which the ecological success of ants as mutualistic defenders repeatedly and independently favored the evolution of EFN-bearing phenotypes across unrelated plant lineages—though the phylogenetic co-occurrence of ant diversification and EFN proliferation cannot by itself establish directionality of causation.

Within angiosperms, EFN distribution is phylogenetically uneven: gains and losses occur across virtually all orders, but certain lineages—notably Fabaceae, Euphorbiaceae, Malvaceae, Passifloraceae, and Salicaceae sensu lato—show disproportionately high EFN richness and, within well-studied groups such as *Senna* (Fabaceae), rapid and repeated evolutionary shifts in EFN position and morphology even at the genus level [3,8,37]. This pattern suggests that clade-specific ecological or developmental factors predispose particular lineages to EFN evolution and that—once the trait arises—its modular, developmentally unconstrained character allows it to be repeatedly refined within a lineage rather than fixed by a single ancestral form. The disproportionate EFN richness of Salicaceae sensu lato is directly relevant to the present review: it positions this family, and *Idesia polycarpa* within it, as a particularly informative phylogenetic context for studying the origin and diversification of indirect defensive traits in woody plants.

### 3.4. Cryptic Morphology and Detection Challenges in Woody Plants

This morphological and phylogenetic complexity underlies a persistent and consequential bias in EFN research: herbaceous species have been disproportionately studied, while trees in particular remain substantially underrepresented in anatomical, chemical, and ecological investigations of EFN biology [3,7]. This apparent underrepresentation is more likely to reflect a mismatch between commonly used detection methods and the cryptic, developmentally dynamic nature of EFNs in these species than true biological scarcity—though the relative contribution of detection bias versus genuine rarity has not been formally quantified. Consistent with this interpretation, the inaugural structural description of EFNs in Sapindaceae required histochemical and microscopic methods to characterize nectaries that are inconspicuous in external morphology [35]—illustrating that even in families where EFN presence was already suspected, structural detection depends on methods beyond visual survey.

Three partially overlapping sources of crypticity underlie this detection bias. First, many EFNs in trees and shrubs are structurally inconspicuous—small, flattened, or parenchymatous in form—and lack the elevated, gland-like appearance that makes EFNs in many herbaceous species readily identifiable by visual survey [7,35]. The prevalence of cryptic EFN types in trees does not imply reduced ecological relevance; it implies that standard visual detection methods may be insufficient. Histochemical staining, scanning electron microscopy, and targeted secretion assays are needed to document EFN presence and activity in woody species reliably, yet these methods are rarely applied at the spatial and temporal scales required for comprehensive forest surveys.

Second, secondary growth progressively alters the surface architecture of woody stems and branches over the life of a tree. Bark formation can physically obscure or permanently cover EFNs that were present on younger stem tissue, rendering them undetectable at the organ ages typically sampled in field studies [7,8]. The apparent absence of EFNs on mature woody stems may therefore reflect burial by secondary tissue rather than genuine absence. This distinction carries direct functional consequences: whether herbivory-induced signaling can trigger secretory responses in bark-occluded EFNs, and whether ants can reliably locate such structures on architecturally complex mature stems, remain open questions that existing studies have not addressed. A further consequence of secondary growth is structural: as trees increase in height, the energetic cost to ants of traversing the stem to reach canopy-level EFNs increases substantially, potentially limiting the effectiveness of ant-mediated defense in tall trees in ways that have no parallel in herbaceous systems [38]. This constraint applies specifically to ground-nesting ants that patrol trees from below; it does not extend to ants permanently resident in the canopy through domatia-based mutualisms, such as *Pseudomyrmex* in *Acacia* or *Azteca* in *Cecropia*, which depend on nesting structures rather than EFN reward and constitute a mechanistically distinct form of ant–plant association outside the present review’s scope. Surveys conducted without accounting for organ age and tree height will therefore underestimate both EFN occurrence and the constraints on its defensive function.

Third, EFN expression in woody plants is frequently seasonal and ontogenetically regulated, adding a temporal dimension to the detection challenge. Secretion activity may be confined to specific phenological windows and decline or cease entirely outside those periods, so that EFNs documented in one season may be morphologically inconspicuous or functionally inactive at other times of the year [35,39]. Ontogenetic regulation adds a further layer: juvenile tissues commonly produce little to no nectar, while secretion intensity increases as organs mature, resulting in pronounced age-dependent variation in EFN activity across the plant body [10]. Studies conducted on young plants, on juvenile tissues, or outside peak secretion periods will therefore systematically underestimate both the prevalence and the functional significance of EFNs in woody species.

The combined effect of these three sources of crypticity is a research record that substantially underrepresents EFN diversity and occurrence in woody plants. Foundational anatomical and developmental mapping—systematically documenting EFN presence, structural form, and ontogenetic trajectory across diverse woody organs and lineages—is consequently a prerequisite for any further progress in this field. Without this baseline, neither the chemical ecology of extrafloral nectar nor the defensive dynamics it underpins can be accurately characterized for these systems.

### 3.5. Synthesis

Characterizing the chemical composition of the secretions these structures produce—a task complicated, in practice, by the logistical demands of accessing tree canopies through climbing, scaffolding, or canopy cranes—is the question to which this review now turns.

## 4. Chemical Ecology of Extrafloral Nectar in Woody Plants

Extrafloral nectar is the proximate medium through which EFN-bearing plants recruit and retain arthropod defenders—and its chemical composition is therefore the central determinant of whether any given EFN system functions as an effective indirect defense. The sugar profile governs foraging behavior and recruitment intensity; the amino acid content sustains resident colonies capable of sustained patrol; and the secondary metabolite suite acts as a selective filter that retains effective mutualists while deterring exploiters [11,21]. However, as the synthesis in Section 4.3 makes explicit, the chemical dimension of woody EFN systems remains the least developed of the three pillars addressed in this review—a necessary foundation for understanding indirect defense, but one for which woody-specific data are currently too sparse to support the level of synthesis achieved for structural distribution (Section 3) and functional efficacy (Section 5). This section examines each of these compositional components in turn, then addresses the biochemical machinery and regulatory mechanisms governing secretion—including the evidence for inducible secretion in woody plants and the critical unresolved questions about its generality and constraints across perennial systems.

### 4.1. Nectar Composition and Mutualist Recruitment

Extrafloral nectar is dominated by the sugars sucrose, glucose, and fructose, but the functional significance of their relative proportions is oriented toward ant mutualist recruitment rather than pollinator attraction [11,21,40]. The specific sugar composition of extrafloral nectar directly modulates ant foraging behavior: the sugar profile influences recruitment intensity, foraging duration, and patrolling frequency, with consequences for the strength and reliability of herbivore deterrence [41,42]. Beyond sugars, extrafloral nectar is characteristically richer in free amino acids than floral nectar. This elevated amino acid content provides a nitrogen source capable of supporting ant colony growth, maintenance, and the metabolic costs of sustained defensive activity—the demands of a resident social colony rather than a transient individual forager [21,22,43,44].

In addition to these primary nutritional components, extrafloral nectar frequently contains secondary metabolites—including phenolics and specific proteins—that serve a qualitatively distinct function. Rather than maximizing attractiveness to all visitors, these compounds act as selective filters: they deter less-effective mutualists and nectar thieves while retaining aggressive ant defenders and additionally suppress microbial spoilage of the nectar resource, thereby stabilizing the mutualism over time [2,45,46]—a dual function demonstrated by González-Teuber et al. [47], who showed that chitinase and β-1,3-glucanase proteins in extrafloral nectar causally inhibit nectar-colonizing fungi, establishing that secondary metabolite composition is functionally tuned to protect the reward rather than merely to attract visitors. Nectar chemistry thus operates simultaneously as a recruitment signal, a nutritional reward, and a partner-filtering mechanism—three functions whose combined effect shapes the composition and quality of the attendant ant community [31,45].

Despite the established importance of all three compositional components, the chemical resolution of extrafloral nectar in woody plants remains limited, constraining our ability to predict ant recruitment and the effectiveness of indirect defense in forest systems [7,31,45]. Empirical data on nectar composition in woody EFN systems remain scarce but suggest important differences from herbaceous models. Morphological and anatomical surveys of woody EFNs, though extensive, have not been paired with quantitative chemical analysis of the nectar itself [7], leaving the compositional counterpart of this well-documented structural diversity effectively unknown (Table 1). Given the pronounced structural diversity already documented among woody EFN types (Section 3), substantial compositional variability among co-occurring taxa is plausible, and—if present—would be likely to influence both the identity and behavior of visiting ant assemblages, particularly in structurally complex forest environments where foraging strategies and competitive interactions are more heterogeneous. The limited availability of comparable compositional datasets across woody taxa therefore represents a critical barrier to linking nectar chemistry with ecological function at the community level and restricts the transferability of trait-based predictions derived from herbaceous systems. This gap is ecologically consequential. In forest ecosystems, the diversity of potential arthropod visitors—including many ineffective or antagonistic species, as well as parasitoid wasps that exploit extrafloral nectar as a food resource in herbaceous systems [48] and likely occur as EFN visitors in woody systems as well, though their occurrence there remains undocumented—substantially exceeds that of most herbaceous study systems [22,43]. It is precisely the chemical signature of extrafloral nectar that filters ant visitor identity and determines mutualist quality under these more complex conditions.

### 4.2. Regulation of Nectar Secretion: Inducibility and Its Complexities in Woody Plants

The compositional properties described above are not fixed attributes but are subject to dynamic regulation in response to both herbivore damage and internal developmental and environmental states. Understanding this regulatory dimension is essential for predicting when EFN-mediated defense will be most active—and it is here that some of the most important and most incompletely resolved questions in this area arise.

At the biochemical level, studies in herbaceous model systems have established that nectar secretion is mediated by the coordinated activity of sucrose phosphate synthases, cell wall invertases, and sugar transporters of the SWEET family, which together govern transmembrane sugar flux from phloem to secretory cells—a mechanism characterized primarily in floral nectaries [50,51] but subsequently shown to operate in EFNs as well [49]. The relative activity of cell wall invertases is particularly significant because it determines the sucrose-to-hexose ratio in the secreted nectar, directly shaping the chemical signal available to ant mutualists—a relationship demonstrated in *Ricinus communis* L. [49,50,51]. This biochemical machinery is also the proximate target of inductive signals: in *R. communis*, jasmonic acid upregulates cell wall invertase activity and increases phloem sugar flux to EFNs, establishing a mechanistic link between wound signaling and nectar output [49]. The light-dependence of jasmonate-mediated induction, demonstrated in lima bean (*Phaseolus lunatus* L.), adds a further regulatory layer—secretion is enhanced only under light conditions coinciding with peak ant foraging activity, suggesting fine-tuned temporal coordination between plant signaling and mutualist behavior [26]. Whether the SWEET transporter repertoire, cell wall invertase isoforms, and jasmonate signaling components that mediate these responses in herbaceous systems are conserved or functionally divergent in perennial trees has not been investigated in the context of EFN secretion—a gap that transcriptomic and metabolomic profiling of secretory tissues across developmental stages is best positioned to address.

A key property of EFN secretion across many plant species is its inducibility: nectar output can be upregulated in response to herbivore damage or wounding, typically mediated by jasmonate signaling, leading to enhanced ant recruitment and improved herbivore deterrence [26]. This inducible response is not confined to herbaceous systems: Heil et al. [52] demonstrated jasmonate-mediated EFN induction in field-grown Macaranga tanarius—a woody pioneer tree—establishing that the underlying signaling pathway operates in at least some woody species. Ness [53] further demonstrated that the woody tree *Catalpa bignonioides* Walter markedly increases extrafloral nectar production following caterpillar herbivory and attracts ant bodyguards in response—confirming that this inducible capacity extends beyond the Macaranga system to phylogenetically distant woody lineages. Evidence from *Populus tremuloides* Michx. further suggests that EFN expression is strongly heritable, with high broad-sense heritability for both constitutive expression (0.74–0.82) and inducibility (0.85), and is inducible in response to defoliation [54].

Two further findings from that study are directly relevant to woody plant EFN biology. First, the high heritability of inducibility provides genetic evidence that EFN-mediated defense in trees has the capacity to respond to evolutionary selection—a prerequisite for indirect defense to function as an adaptive trait in long-lived perennials. Second, the study documents a pronounced age-dependent decline in EFN density: one-year-old trees had over 50% greater EFN density than trees aged ten years or older [54], providing empirical support for the role of ontogenetic regulation in shaping the expression and inducibility of indirect defense. Earlier work on *Catalpa speciosa* Warder ex Engelm. further showed that EFN-bearing trees experience reduced herbivory and enhanced fruit production relative to plants without ant attendance [55], confirming that ant-mediated defense translates into measurable fitness benefits in at least some woody species. Taken together, these studies—spanning two genera and three independent lines of evidence—demonstrate that herbivory-inducible EFN secretion and its downstream defensive consequences are not confined to herbaceous systems; they do not, however, establish the generality of this capacity across the phylogenetic and ontogenetic breadth of woody plants.

Whether mature trees broadly retain the acute jasmonate-mediated inducible response documented in herbaceous systems—or whether anatomical and physiological constraints associated with secondary growth and extended ontogeny limit this capacity in some species or organ types—remains an open and pressing question [10,27]. Ontogenetic regulation adds further complexity: juvenile tissues commonly produce little or no nectar, while secretion intensity typically increases with organ maturity, generating pronounced within-plant heterogeneity in defense allocation across developmental stages [10]. Seasonal dynamics compound this further—nectar secretion rates and chemical profiles may fluctuate substantially with climatic cycles and competing demands on carbon and nitrogen allocation, potentially decoupling EFN activity from periods of peak herbivore pressure [30,56,57]. The result is a spatiotemporally dynamic nectar landscape within individual trees, shaped by the interaction of herbivore-induced, ontogenetic, and seasonal regulatory signals—a complexity that current models, developed largely in herbaceous systems, are not designed to capture [8,9].

### 4.3. Synthesis

The chemical ecology of extrafloral nectar—its compositional components and regulatory dynamics—constitutes the proximate mechanism linking EFN structure to defensive outcome. Evidence from *Catalpa* and *Populus* supports that the biochemical machinery linking herbivory perception to nectar upregulation is operational in at least some woody species [53,54,55]. Whether the resultant nectar signal is compositionally sufficient to recruit and retain effective ant mutualists under natural forest conditions, however, depends on factors—sugar profile, amino acid content, and secondary metabolite suite—that remain largely uncharacterized across woody lineages. Resolving this compositional gap will be essential for determining whether nectar chemistry translates into effective defensive outcomes in forest trees. This review accordingly treats woody EFN chemistry as a necessary but currently underdeveloped foundation and positions the structural and functional advances detailed in Section 3 and Section 5—rather than chemical characterization—as its primary contributions.

## 5. EFN-Mediated Indirect Defense: Evidence, Efficacy, and Ecological Scaling

Whether EFN-mediated ant attendance actually reduces herbivory and enhances plant fitness—and under what conditions this defense can be relied upon—is the empirical question on which the ecological significance of EFNs ultimately rests. The evidence is broadly affirmative but inherently conditional: defensive outcomes vary substantially across plant species, ant assemblages, herbivore communities, and environmental contexts in ways that are not incidental but structured by identifiable ecological factors. This conditionality is particularly pronounced in long-lived woody plants, where ontogenetic complexity, canopy architecture, and temporal variability in nectar secretion generate a dynamic defensive landscape with no direct parallel in the annual or short-lived systems where EFN function has been most thoroughly characterized. This section examines the evidence for defensive efficacy, the factors governing its reliability, and the challenge of scaling individual-level interactions to community and ecosystem dynamics in forest systems.

### 5.1. Evidence for Defensive Efficacy: From Herbaceous Systems to Woody Plants

The defensive function of EFNs is experimentally well-supported across diverse plant–ant systems. Meta-analyses confirm that ant attendance significantly reduces herbivory and enhances plant fitness, and manipulative experiments—ant-exclusion trials and nectar augmentation assays, dating back to some of the earliest documented tests of this kind [58]—have established direct causal links between extrafloral nectar rewards and defensive outcomes [5,59,60]. Trager et al. [61], synthesizing data across 59 ant–plant species pairs, quantified ant presence as reducing herbivory by 62% and increasing plant reproductive output by 49% on average—providing a quantitative benchmark for the magnitude of indirect defense that EFN-bearing plants can achieve under effective ant mutualism; comparative field data from tropical systems similarly confirm lower herbivory rates in EFN-bearing species relative to co-occurring plants without EFNs [62]. This evidence base, however, derives overwhelmingly from herbaceous systems, where EFNs are morphologically accessible and experimentally tractable.

Direct evidence from trees is more limited but consistent with the same pattern. Stephenson [55] provided one of the earliest demonstrations specifically of EFN-mediated (rather than domatia-mediated) indirect defense in a woody species, showing in *Catalpa speciosa* that trees with ant-attended EFNs experienced significantly lower caterpillar herbivory and produced more than twice the fruit set of trees from which ants were experimentally excluded—establishing that EFN-mediated defense translates into quantifiable fitness benefits in a long-lived woody species. Critically, the inducibility of EFN secretion—a hallmark of indirect defense in herbaceous systems—produces measurable defensive outcomes in woody species: herbivory-induced nectar upregulation in *Catalpa bignonioides* results in active ant bodyguard recruitment [53], and *Populus tremuloides* exhibits genetically based, herbivory-inducible EFN expression with documented ontogenetic regulation [54]. Together, these cases demonstrate that herbivory-inducible secretion is ecologically operative—producing the ant recruitment and herbivore deterrence on which indirect defense depends. Nevertheless, available evidence from woody species remains taxonomically and ecologically limited, constraining generalization across the phylogenetic breadth of EFN-bearing trees and shrubs.

### 5.2. Context-Dependence and Variation in Defensive Outcomes

The defensive efficacy of EFN-mediated mutualisms is not absolute. Outcomes vary substantially across plant species, ant assemblages, herbivore communities, and environmental contexts, and this variation is not random but structured by identifiable ecological factors [38,63].

The identity and behavioral ecology of the attendant ant species are primary determinants of defensive quality. Ant species vary markedly in aggressiveness, colony size, recruitment efficiency, and territoriality—traits that translate directly into differences in herbivore deterrence capacity. Lanan and Bronstein [38] demonstrated this at the colony level, showing that recruitment of *Crematogaster opuntiae* to EFN-bearing cacti was spatially non-independent among plants up to 5 m apart, with colony territory structure governing which plants received consistent ant attendance—a finding that shifts the unit of analysis from individual plant reward to colony-scale foraging decisions and has direct implications for predicting defensive coverage in structurally complex woody systems. The same EFN-bearing plant may therefore receive substantially different levels of protection depending on which ant species dominate locally. Defensive efficacy can also depend on the spatial scale at which it is measured: in *Populus tremuloides*, EFN expression had no detectable effect on leaf-level damage by a specialist leaf miner, yet at the whole-plant or branch scale, higher EFN expression was associated with greater predation of the miner by patrolling ants and correspondingly reduced damage—indicating that the same defensive interaction can appear absent or effective depending on the scale of observation [64]. The broader biotic and abiotic environment modulates interaction strength further—Aranda-Rickert et al. [63] showed that water stress simultaneously suppressed nectar secretion and ant visitation rates, demonstrating that abiotic conditions can constrain multiple components of the mutualism in concert. Nectar availability from alternative sources, habitat structure, and microclimatic heterogeneity all shape ant foraging decisions and competitive dynamics, affecting the consistency of ant attendance on EFN-bearing plants. The composition of the herbivore community itself also shapes outcomes, as ant effectiveness varies across herbivore guilds—ants are generally more effective against small, soft-bodied insects such as caterpillars than against large or mobile beetles [65]; moreover, ants themselves often constitute well under half of all arthropods visiting woody EFNs, with flies and other non-ant taxa forming a substantial and ecologically heterogeneous component of the visitor assemblage [22,66].

Context-dependence is particularly pronounced in long-lived woody plants. The spatiotemporally dynamic nectar landscape generated by the interaction of ontogenetic, seasonal, and herbivory-induced regulatory signals introduces a further dimension of variability that has no direct parallel in herbaceous systems: within-canopy heterogeneity in light, microclimate, and ant accessibility means that the same tree can simultaneously present high-quality EFN reward at some organs and negligible secretion at others [10,30,57]. The consequence is that EFN-mediated defense in forest trees is inherently conditional—effective under some combinations of plant developmental stage, ant community composition, and environmental conditions, but unreliable or absent under others [67].

### 5.3. From Individual Defense to Community and Ecosystem Dynamics

EFN-mediated interactions do not operate in isolation at the level of individual plants but aggregate to shape ant assemblage structure, plant–herbivore dynamics, and trophic network organization at the community and ecosystem levels. Understanding these scaling effects is essential for characterizing the full ecological significance of EFNs in forest systems—and it is at this scale that trees present both the greatest complexity and the greatest research gaps.

At the within-plant scale, the spatial arrangement of EFNs guides ant patrolling patterns across the plant body, concentrating defensive activity on the most vulnerable tissues in a manner consistent with optimal defense theory [34,68]. At the between-plant scale, EFN-mediated interactions can generate associational resistance: ant species recruited to EFN-bearing plants extend their patrolling activity to neighboring vegetation, reducing herbivory on plants that do not themselves bear EFNs [27,69,70]. This effect has been demonstrated experimentally in woody systems at two complementary levels of resolution. Moura and Del-Claro [71] showed that plants supported by EFN-bearing woody neighbors attracted higher ant species richness and visitation rates and experienced nearly three-fold lower leaf herbivory than unsupported plants, with artificial nectar supplementation increasing ant visitation on neighboring plants by 2.5-fold—establishing a direct causal link between extrafloral nectar availability and the spatial extent of indirect defense within a plant community. Complementing this, Staab et al. [72] demonstrated the same effect among true forest trees in a replicated diversity experiment: trees adjacent to EFN-bearing species harbored higher ant biomass and species richness, experienced lower caterpillar biomass, and showed altered leaf defense trait composition, with non-EFN trees potentially reallocating saved defense investment toward growth. Together, these studies establish that associational resistance mediated by EFN-bearing trees is not a theoretical extrapolation from herbaceous systems but an empirically validated phenomenon operating among trees in forest ecosystems, with consequences extending beyond herbivore suppression to influence defense allocation and growth of neighboring vegetation.

These community- and ecosystem-level dynamics remain poorly understood in forest systems relative to their ecological importance. The structural complexity of forest trees—deep canopies, persistent woody architecture, and long-lived tissues—introduces layers of spatial and temporal heterogeneity in EFN expression and ant activity that make community-level patterns difficult to document and interpret mechanistically [7,8,10]. Most existing evidence for EFN effects on ant community structure and associational resistance derives from herbaceous or structurally simple systems; how these dynamics play out among architecturally complex trees, where ant foraging ranges, canopy connectivity, and within-tree EFN heterogeneity all interact, remains largely unexplored.

### 5.4. Synthesis

EFN-mediated indirect defense is an experimentally validated strategy in woody plants, but its efficacy is inherently conditional—shaped by ant species identity, herbivore community composition, and the ontogenetic, seasonal, and architectural complexity that distinguishes perennial trees from the annual or short-lived systems in which EFN function has been most thoroughly studied. Crucially, EFN-mediated interactions do not operate at the level of individual plants alone: their capacity to generate associational resistance among neighboring trees and to shape multi-trophic network structure at the community level makes them ecologically consequential at scales that individual-level studies cannot capture. These community-scale dynamics in forest ecosystems remain among the least characterized aspects of tree EFN biology, and addressing them requires a model system capable of integrating anatomical, chemical, and ecological investigation within a single coherent framework—one that combines the biological complexity of a perennial tree with the practical tractability needed for manipulative experimentation at multiple scales. *Idesia polycarpa*, proposed in the following section, satisfies these criteria.

**Figure 1:**
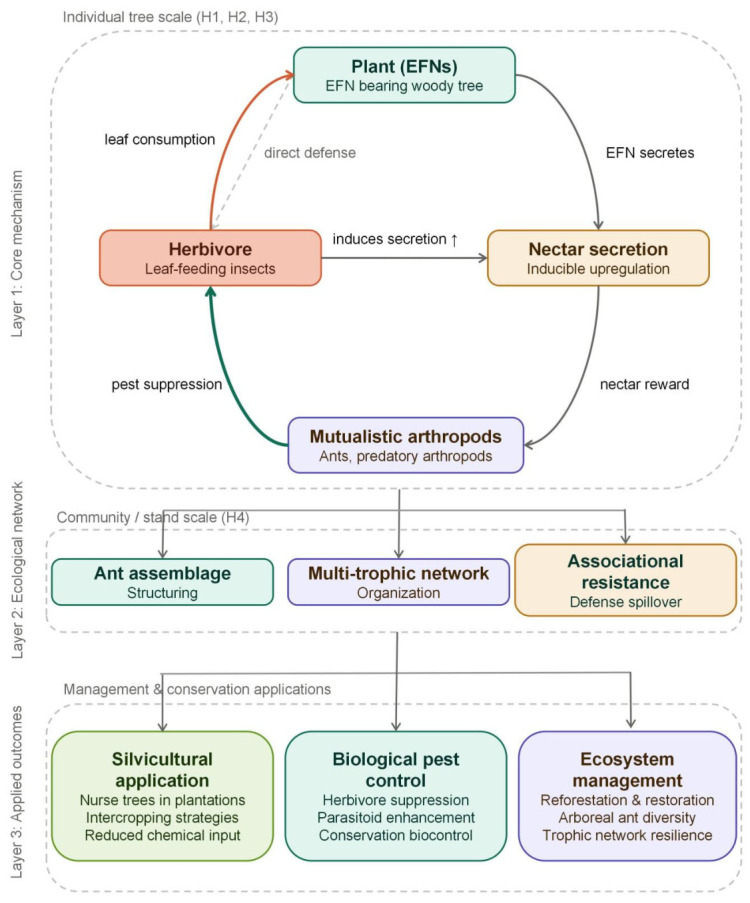
Conceptual framework illustrating the role of extrafloral nectaries (EFNs) in forest ecosystems across three scales. Layer 1 (individual tree scale) shows the core indirect defense mechanism linking herbivory, nectar secretion, and arthropod-mediated herbivore suppression, with constitutive direct chemical defense operating in parallel. Layer 2 (community/stand scale) shows the ecological network effects that emerge from the aggregation of EFN-mediated interactions, including ant assemblage structuring, multi-trophic network organization, and associational resistance. Layer 3 (management and conservation scale) shows the applied outcomes informed by Layer 2 processes, encompassing silvicultural integration of EFN-bearing species, biological pest control, and ecosystem restoration and monitoring. Solid black arrows indicate direct ecological interactions; the dashed gray arrow indicates constitutive direct chemical defense; the solid green arrow indicates indirect defense outcome; Layer 1–2 and Layer 2–3 transitions represent many-to-many relationships among nodes. Four testable hypotheses derived from this framework are mapped onto the layers where they operate (H1–H3: Layer 1; H4: Layer 2) and are developed in full in Section 7.2. H1, detection bias in woody EFN occurrence; H2, ontogenetic decline in jasmonate-mediated inducibility; H3, partitioning between direct and indirect defense strategies; H4, density-dependent associational resistance at the community scale.

The three-layered framework developed across Section 3, Section 4 and Section 5—spanning the core defensive mechanism at the individual tree scale, the ecological network effects at the community scale, and the management and conservation applications that follow from them—is summarized in Figure 1.

## 6. *Idesia polycarpa* as a Candidate Model System for Woody Plant EFN Research

The three preceding sections have collectively established what is needed to advance the study of EFN biology in woody plants: systematic anatomical and developmental characterization of EFN structure and distribution, comprehensive chemical profiling of extrafloral nectar composition, experimental quantification of inducible secretion across ontogenetic stages and seasons, and scaling of individual-level defensive interactions to community and ecosystem dynamics. Fulfilling this research agenda requires a study system that simultaneously embodies the biological complexity of perennial trees and remains practically tractable for integrated anatomical, chemical, and ecological investigation. *Idesia polycarpa* is proposed here as a well-supported candidate—not as a species already well-characterized in EFN research, but as one whose combination of confirmed secretory activity, documented ant visitation, genomic resources, and phylogenetic context makes it a well-justified starting point for developing an integrated EFN research program in temperate deciduous trees. As Table 2 makes explicit, this candidacy reflects a deliberate trade-off: *I. polycarpa* currently lags behind *Macaranga*, *Catalpa*, and *Populus* on every dimension of characterization but offers a phylogenetically and genomically tractable starting point for closing that gap. The evolutionary lability of EFNs—whereby presence, position, and structure can vary markedly even within a genus—does not diminish the utility of a model system; rather, it makes one necessary. *I. polycarpa* is not proposed as a representative of the full breadth of EFN diversity across woody angiosperms but as an experimentally tractable reference point from which comparative questions can be systematically extended to other woody lineages.

### 6.1. Phylogenetic Position and Botanical Context

*Idesia polycarpa* is placed within Salicaceae sensu lato [73,74], closely related to the well-studied model genera *Populus* and *Salix* and occupying an intermediate phylogenetic position within the family [74,75]. This placement has direct implications for EFN research on two grounds.

First, Salicaceae sensu lato is among the angiosperm lineages showing disproportionately high EFN richness [3,8], making *I. polycarpa* an informative entry point for understanding EFN diversification within a woody clade with a documented history of indirect defense evolution. Second, the phylogenetic proximity of *I. polycarpa* to *Populus*—for which genomic resources are well-developed and for which EFN-mediated indirect defense, including its inducible and heritable dimensions, has been experimentally characterized in *Populus tremuloides* [54]—creates opportunities for comparative molecular work that would be unavailable in a more phylogenetically isolated model. The nuclear genome of *I. polycarpa* has recently been sequenced and assembled at the chromosome level—comprising approximately 1.21 Gb across 21 pseudochromosomes with 42,086 annotated protein-coding genes [75]—providing a robust genomic foundation for transcriptomic, metabolomic, and comparative analyses central to EFN research. This assembly-based estimate falls between two independent preliminary estimates reported in the same study (flow cytometry: ~1.03 Gb; k-mer analysis: ~1.23 Gb), illustrating the sensitivity of genome size estimation to assembly method even within a single study; comparable cross-method variation has been reported for the reference genome of *Populus trichocarpa* itself, for which published estimates range from approximately 380 Mb to 690 Mb depending on sequencing approach and assembly version, with ~500 Mb being the figure most commonly cited. Whether the resulting size difference between the two species primarily reflects differences in repetitive sequence content or other factors has not been directly assessed in either source and remains an open question. Its botanical attributes further support practical suitability: the deciduous habit, moderate stature of 8–21 m, and accessible canopy architecture facilitate both controlled experimentation and repeated field sampling across developmental stages and seasons.

### 6.2. EFN Phenotype: Known Evidence and Critical Knowledge Gaps

**Figure 2:**
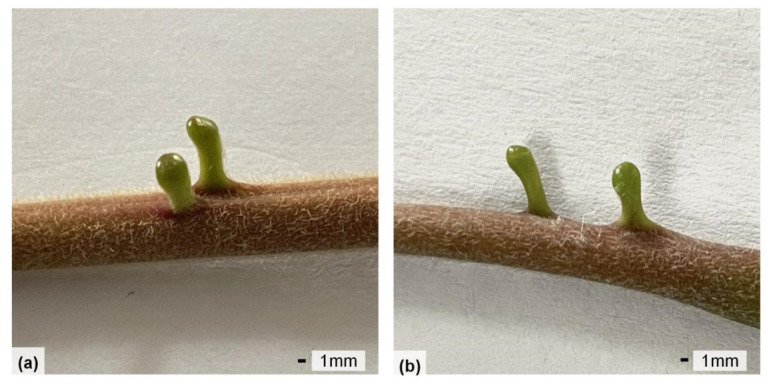
Petiolar secretory glandular structures of *Idesia polycarpa* under laboratory conditions. (**a**) Two glandular structures arranged side by side at the same petiolar node, viewed from the front; note surface liquid accumulation at the apex of both structures. (**b**) Two glandular structures arranged in tandem along the petiolar axis at the same node, viewed from the side. Scale bars = 1 mm. Petiolar glandular structures measure approximately 2–3 mm in diameter and 5–10 mm in height. These images document the physical occurrence and morphology of the glandular structures under controlled conditions and are not intended as evidence of secretory chemistry or defensive function, which are addressed separately in Section 6.2 and Section 6.3.

**Figure 3:**
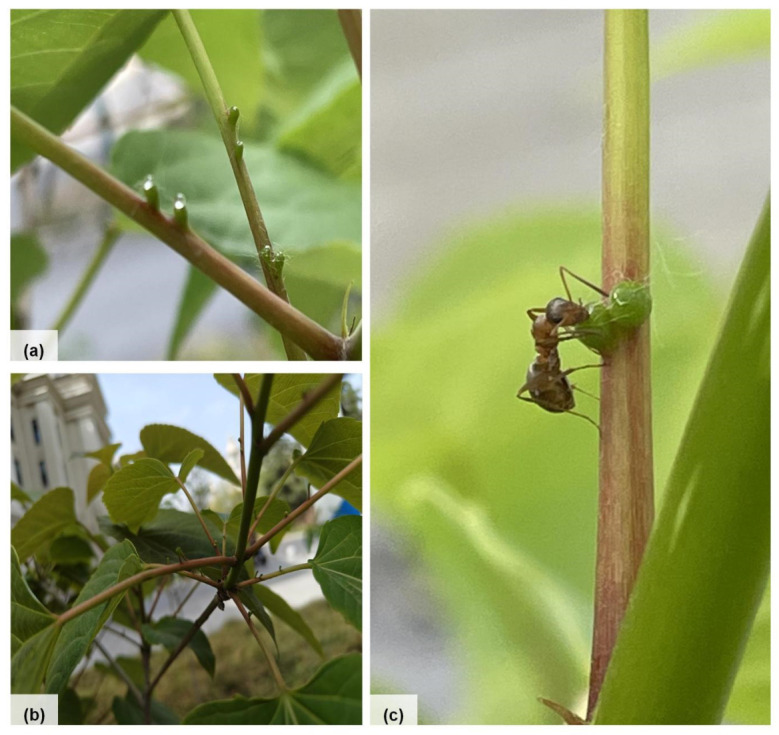
Field evidence of secretory activity, spatial distribution, and ant visitation at petiolar glandular structures of *Idesia polycarpa*. (**a**) Visible liquid droplets accumulating at the apices of petiolar glandular structures under natural field conditions, confirming active secretion. (**b**) Systematic occurrence of petiolar glandular structures at multiple nodes along a single branch of a field-grown individual, consistent with the spatial logic of optimal defense theory in which secretory structures are concentrated on herbivore-vulnerable vegetative tissues. (**c**) An ant individual in direct feeding contact with a petiolar glandular structure, with mouthparts positioned at the gland apex; a second glandular structure at the same petiolar node is visible to the left. Taxonomic identification of this ant to genus or species was not possible from field photographs alone, as no voucher specimen was collected during this observational survey; formal identification would require specimen collection and expert taxonomic examination. No scale bars in (**a**–**c**); glandular structures are approximately 2–3 mm in diameter and 5–10 mm in height, comparable to those shown in Figure 2. These photographs document field-observed secretion and ant visitation; they are presented as observational evidence motivating the candidacy of *I. polycarpa* as a model system, not as experimental confirmation of nectar chemistry or defensive efficacy, both of which remain to be formally tested.

*Idesia polycarpa* bears morphologically identifiable glandular structures on vegetative organs, particularly on leaf petioles and at gland-tipped leaf margin teeth [22]. The petiolar structures are morphologically prominent, measuring approximately 2–3 mm in diameter and 5–10 mm in height, with an elevated capitate form readily distinguishable from surrounding petiolar tissue without histochemical assistance—a structural conspicuousness that contrasts with the cryptic EFN types prevalent in many other woody species [3,7,35] and that renders *I. polycarpa* particularly tractable for anatomical and secretory investigation (Figure 2a,b); their systematic occurrence across multiple petiolar nodes of field-grown individuals further reflects the spatial logic of optimal defense theory (Figure 3b). Their structural similarity to glandular organs documented on the petioles and leaf margins of confirmed EFN-bearing Salicaceae members is reinforced by direct field observations: these structures actively secrete liquid, with droplets accumulating visibly at gland apices under natural conditions (Figure 3a), and ant individuals have been photographically documented visiting the secretory structures with feeding contact behavior (Figure 3c). The limits of current evidence must nonetheless be stated precisely: the chemical composition of the secretion has not been formally quantified, and whether ant visitation translates into measurable herbivore deterrence and plant fitness benefits remains to be experimentally established. Preliminary metabolomic and volatile profiling of *I. polycarpa* petiole glands is currently underway in our laboratory [76], comparing gland tissue with adjacent leaf and shoot tissue using UPLC-MS/MS and GC-MS platforms, respectively. This ongoing work is intended to establish the chemical baseline that Section 4 identifies as currently lacking for this species, though it characterizes gland tissue composition rather than the secreted nectar itself, leaving the latter as a distinct and still-unaddressed empirical gap. *I. polycarpa* thus represents a partially characterized system—one with confirmed secretory activity and documented ant association, but whose full EFN functional status awaits experimental validation.

What makes this gap particularly tractable is that *I. polycarpa* has an established phytochemical context within which the question of indirect defense can be directly situated. Phytochemical investigation of its leaves has revealed nine previously undescribed phenolic natural products structurally related to salicinoids—the characteristic direct defense compounds of *Populus* and *Salix*—and feeding experiments with larvae of *Lymantria dispar* (L.) (a broadleaf generalist) and *Cerura vinula* (L.) (a Salicaceae specialist) demonstrated significantly lower survival rates and mass gain on an *I. polycarpa* leaf diet compared to *Populus nigra* L. [77]. These findings establish two facts directly relevant to the research case for *I. polycarpa*. First, the species is a viable and toxic host for both a specialist and a generalist lepidopteran herbivore under controlled laboratory feeding conditions, demonstrating that the chemical and nutritional basis for herbivore pressure is present; whether this translates into comparable herbivore pressure under field conditions, where host choice, natural enemy activity, and phenological timing all modulate realized damage, has not been directly assessed and remains an assumption rather than an established fact. Second, *I. polycarpa* has demonstrably invested in direct chemical defense through salicinoid-related compounds, establishing that it operates within the broader Salicaceae chemical defense framework and that its defense biology is tractable with existing phytochemical methods. These two facts do not themselves indicate whether indirect defense is deployed; they define the ecological and chemical context within which the question of EFN function is most productively posed. Whether *I. polycarpa* additionally deploys EFN-mediated indirect defense—and if so, how direct and indirect strategies interact and partition costs across developmental stages and herbivore guilds—remains entirely open and constitutes the primary empirical question motivating its proposal as a model system. The integration of direct and indirect defense within a single phylogenetically and chemically well-characterized woody species is a research opportunity that has rarely been systematically pursued [78].

Available evidence further suggests that glandular structure expression in *I. polycarpa* may vary with ontogenetic stage and season [22], offering a natural developmental gradient along which to investigate the spatiotemporal heterogeneity in defense allocation—among the least understood features of indirect defense in long-lived plants.

### 6.3. Research Potential of Idesia polycarpa for Woody EFN Studies

*Idesia polycarpa* is well-positioned to support the research priorities identified in Section 3, Section 4 and Section 5. Its morphologically accessible active secretory glandular structures overcome the detection challenge that afflicts many woody species. Its moderate stature, accessible canopy, and deciduous phenology facilitate repeated sampling across developmental stages and seasons—enabling both the comprehensive chemical profiling and the manipulative experimentation that perennial EFN systems have lacked. Its established salicinoid profile, chromosome-level genome, and phylogenetic proximity to *Populus* provide the mechanistic resolution needed to link EFN traits to defensive outcomes with unusual precision [27,28]. Its occurrence in native East Asian forest communities allows findings to scale to the community level [72]. The case for *I. polycarpa* rests not on fully demonstrated EFN function—which remains to be established—but on the convergence of confirmed secretory activity, documented ant visitation, genomic resources, and chemical context that make it, among temperate deciduous trees, the most tractable entry point currently available. The specific research priorities this system is positioned to address are detailed in Section 7.2.

## 7. Synthesis and Conclusions

### 7.1. Integrative Synthesis

Across the three dimensions examined in this review—structural, chemical, and functional—a single cross-cutting pattern emerges: detection failures at the structural level determine which EFN systems enter the chemical literature at all, producing a chemical record that is not merely incomplete but systematically biased toward the detectable subset of woody EFN diversity; chemical incompleteness in turn constrains functional studies, which can only assess defensive efficacy in systems where nectar composition has been characterized; and both gaps together limit what can be known about community- and ecosystem-scale dynamics, which depend on mechanistic grounding that neither structural nor chemical research has yet provided for most woody lineages. This is not a set of parallel deficiencies but a propagating one, and resolving these interconnected gaps will require integrated anatomical, chemical, and ecological investigations conducted within tractable woody plant systems. *Idesia polycarpa*—with its confirmed secretory activity, documented ant visitation, and available genomic resources—represents the most tractable entry point currently available through which such questions may be addressed.

### 7.2. Research Priorities

At the structural level, the three compounding sources of detection bias identified in Section 3.4—cryptic morphology, secondary growth, and ontogenetic regulation—generate a first hypothesis: that histochemical and fine-structural surveys of woody species in which EFNs have not been visually recorded will reveal functionally active secretory structures in a substantial proportion of surveyed taxa, consistent with the hypothesis that current underrepresentation reflects methodological limitation more than genuine biological scarcity [3,7,35]. At the regulatory level, there is evidence that jasmonate-mediated inducibility is operational in some woody species, but the generality of this capacity across ontogenetic stages remains unresolved (Section 4.2); together, this generates a second prediction: that EFN secretory inducibility declines with increasing tree age and secondary growth, such that mature trees exhibit a lower amplitude or slower kinetics of nectar upregulation in response to herbivory or exogenous jasmonate application than juvenile trees—a prediction directly testable across developmental stages in *I. polycarpa* and other woody taxa with confirmed EFN activity [10,27,54]. At the functional level, the co-occurrence in Salicaceae woody plants of salicinoid-based direct chemical defense and EFN-mediated indirect defense suggests a third prediction: that the two strategies are functionally complementary rather than redundant, partitioning defensive investment across herbivore guilds and leaf developmental stages in a manner that minimizes total allocation costs—a prediction now tractable in *I. polycarpa*, given the confirmed secretory activity of its petiolar glandular structures and documented ant visitation, pending formal chemical characterization and ant-exclusion experiments [77,78]. Finally, scaling to the community level, the demonstrated capacity of EFN-bearing trees to enhance ant diversity and reduce herbivory on neighboring non-EFN trees [71,72] generates a fourth prediction: that this effect is non-linear with respect to the proportion of EFN-bearing trees in a stand, with threshold densities below which associational resistance collapses—a prediction whose confirmation would establish EFN-bearing trees as keystone mutualists in forest ecosystems.

**Figure 4:**
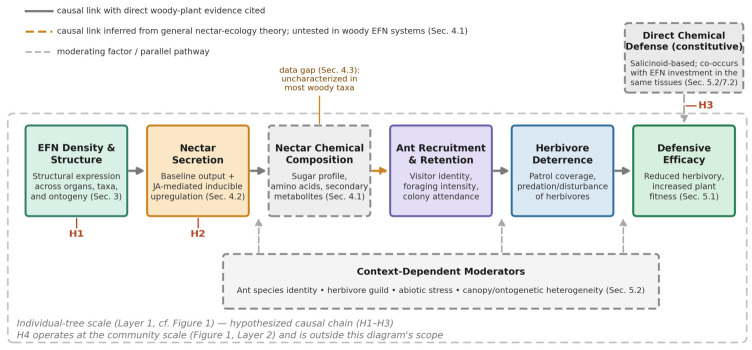
Hypothesized causal chain linking EFN density to defensive efficacy at the individual-tree scale (Layer 1 of Figure 1), elaborating hypotheses H1–H3 (Table 3) into an explicit, testable sequence. Solid gray arrows indicate causal links for which direct evidence from at least one woody species is cited in this review (Section 4.2 and Section 5.1); the amber dashed arrow indicates a link supported by general nectar-ecology theory (Section 4.1) but not yet tested for woody EFN systems, reflecting the chemical-composition data gap identified in Section 4.3. Gray dashed arrows indicate the context-dependent moderators described in Section 5.2, which act on nectar secretion, ant recruitment quality, and the reliability of the deterrence-to-efficacy transition. The parallel constitutive direct-defense pathway (Section 5.2 and Section 7.2) is shown as an independent input converging on defensive efficacy, corresponding to hypothesis H3, which concerns the relative contribution of the two strategies rather than a property of either pathway alone. H4 (density-dependent associational resistance) operates at the community scale (Layer 2, Figure 1) and is outside the scope of this individual-tree diagram.

Figure 4 renders the individual-tree-scale hypotheses (H1–H3) as an explicit causal chain from EFN density to defensive efficacy, distinguishing links supported by direct woody-plant evidence from those currently inferred by analogy with better-characterized herbaceous systems.

Table 3 formalizes these four hypotheses, specifying for each the key variables, the direction of the predicted relationship, and the system and method by which it can be tested, so as to state the framework’s predictive content explicitly rather than leave it embedded in narrative form.

Advancing the field requires translating these predictions into empirical results through progress on four interlinked research priorities. First, systematic anatomical and developmental mapping must document EFN presence, structural diversity, and ontogenetic trajectories across woody organs and lineages—the baseline without which chemical and functional characterization cannot be accurately designed. This mapping must span the full ontogenetic trajectory from juvenile to mature tissue and be replicated across seasons. Phylogenetically informed comparative sampling—pairing EFN-bearing species with their closest EFN-free relatives within EFN-rich lineages such as Salicaceae and Fabaceae—would additionally identify the structural correlates of EFN gain and loss across woody clades [37].

Second, comprehensive chemical profiling must characterize extrafloral nectar composition across sugar ratios, amino acid content, and secondary metabolite suites in woody species and link this chemical signature to ant recruitment, partner fidelity, and defensive effectiveness under natural forest conditions. Profiling must be conducted across developmental stages, diel cycles, and seasons to capture compositional variation that static single-sample approaches cannot detect.

Third, observational and chemical studies must be coupled with experimental manipulation—ant-exclusion trials, nectar augmentation assays, and jasmonate-based induction experiments across developmental stages—to establish causal relationships between EFN traits, ant activity, and plant fitness in genuinely perennial systems. Long-term exclusion experiments spanning multiple growing seasons are particularly needed to quantify the cumulative fitness consequences of indirect defense in trees, where costs and benefits accrue over years rather than weeks and may vary significantly from one year to the next.

Fourth, research must scale to community and ecosystem levels, examining how EFN-mediated interactions shape arboreal ant assemblage structure, generate associational resistance, and contribute to multi-trophic network resilience. Plot-level experiments manipulating the proportion and spatial arrangement of EFN-bearing trees, combined with interaction network approaches across whole forest stands, would reveal whether EFN-bearing trees function as keystone mutualists whose presence disproportionately structures ant diversity and herbivore suppression [71,72].

These four priorities are not independent: anatomical mapping enables targeted chemical sampling; chemical characterization informs the design of meaningful manipulative experiments; and experimental results provide the mechanistic grounding needed to interpret community-level patterns. Progress along all dimensions simultaneously—as an integrated program rather than a series of isolated studies—is what *Idesia polycarpa*, with its confirmed secretory activity, documented ant visitation, and available genomic resources, is positioned to enable.

### 7.3. Applied Implications

The research agenda outlined above carries direct implications for forest management in three directions that are particularly tractable given current knowledge.

EFN biology provides a mechanistic basis for biological pest management in plantation and managed forestry. EFN-bearing species that recruit aggressive ant assemblages could function as nurse trees or intercropping components, suppressing pests through both direct protection and associational resistance extended to neighboring plants [27,79]. This dual potential is supported by evidence across systems: EFN-bearing trees have extended protection to neighboring non-EFN trees in experimental forest plots [72] and to neighboring crop plants in coffee agroforestry [80,81], while directly reducing pest damage on EFN-bearing fruit trees in managed orchards [82]—indicating that this is a biologically grounded strategy rather than a theoretical proposition.

A second direction concerns species selection for reforestation and restoration. Prioritizing EFN-bearing species in mixed plantings could enhance indirect defense capacity, support arboreal ant diversity, and strengthen multi-trophic network complexity in restored stands [27,60,71], with benefits potentially extending to neighboring non-EFN species [72].

A third, more speculative direction follows from the premise that EFN-bearing trees function as ecological nodes that concentrate ant diversity and herbivore suppression across neighboring vegetation [71,72]: if so, disruption of these interactions by habitat degradation, fragmentation, or ant diversity loss may register in ant community composition and EFN visitation rates before the resulting changes become apparent at the broader ecosystem level [69,83]. Whether this offers practical advantages over existing biotic indicators remains untested.

### 7.4. Conclusions

By integrating the structural, chemical, and functional dimensions of EFN biology within a framework explicitly designed for woody plants, and by identifying *Idesia polycarpa* as a candidate model system to anchor and advance this framework, this review charts a course from the current fragmented understanding of forest indirect defense toward a coherent, predictive account of how trees bearing EFN-like structures function within and shape the multi-trophic networks of forest ecosystems. Realizing this potential requires coordinated effort across structural botany, chemical ecology, forest entomology, and conservation management. The stakes are both fundamental and applied: resolving how trees regulate, deploy, and sustain indirect defense across decades of growth will advance core questions in plant defense evolution and multi-trophic ecology, while simultaneously providing the mechanistic foundation needed to incorporate indirect defense traits into evidence-based forest management.

## Figures and Tables

**Table 1:** Illustrative comparison of extrafloral nectar chemical-composition characterization depth between representative well-studied systems and the woody EFN systems addressed in this review (anchored on the structural survey of Machado et al. [7]), highlighting the current data gap for *Idesia polycarpa*.

Compositional Component	Representative Well-Characterized System(s)	Woody EFN Systems—Machado et al. [7]	*I. polycarpa*
Sugar profile (sucrose:hexose ratio)	Cell-wall-invertase-mediated regulation resolved in *Ricinus communis* [49]; broader mechanism (SPS, SWEET) characterized in other floral/model systems [50,51]	Uncharacterized—no chemical analysis conducted [7]	Uncharacterized
Amino acid content	General elevated pattern (vs. floral nectar) documented in nectar-chemistry reviews [21]; no single herbaceous exemplar quantified in this review’s citation set	Uncharacterized—no chemical analysis conducted [7]	Uncharacterized
Secondary metabolites (antimicrobial proteins, phenolics)	Chitinase/β-1,3-glucanase antifungal proteins demonstrated in Acacia EFN—a woody/shrub system, cited here for its resolved mechanism [47]	Uncharacterized—no chemical analysis conducted [7]	Uncharacterized

**Table 2:** Comparative summary of chemical, structural, and functional characterization across four woody EFN model systems. *I. polycarpa* is positioned as a candidate system with confirmed structural and observational evidence, but, unlike *Macaranga*, *Catalpa*, and *Populus*, it currently lacks experimental defense validation.

System	EFN Structure Characterized	Nectar Chemistry Characterized	Inducibility Demonstrated	Functional/Defense Validation
*Macaranga tanarius*	Yes—well-described	Yes	Yes—jasmonate-mediated induction [52]	Yes—ant-mediated indirect defense experimentally confirmed
*Catalpa* (*C. bignonioides*, *C. speciosa*)	Yes—well-described	Partial	Yes—herbivory-induced nectar upregulation and ant recruitment [53]	Yes—ant-exclusion experiments link EFN defense to fitness outcomes, including >2-fold fruit set difference [55]
*Populus tremuloides*	Yes—well-described	Partial	Yes—high heritability (0.74–0.85) for constitutive and induced expression [54]	Partial—heritability and inducibility demonstrated; community-scale defensive efficacy not directly tested in this species
*Idesia polycarpa* (this review)	Partially characterized—secretory activity and morphology confirmed (Section 6.1 and Section 6.2)	Uncharacterized	Not yet tested	Not yet tested—ant visitation documented observationally; no exclusion or fitness experiments to date

**Table 3:** Summary of the four testable hypotheses (H1–H4) generated by the integrated framework, specifying for each the level at which it operates, the key variables involved, the direction of the predicted relationship, and a proposed system and method for empirical testing. H1–H3 operate at the level of the individual tree (Layer 1, Figure 1); H4 operates at the community scale (Layer 2, Figure 1).

Hypothesis	Level	Key Variable(s)	Testable Prediction (Direction Specified)	Proposed System/Method
H1—Detection bias	Structural (Layer 1)	Detection method (visual survey vs. histochemical/SEM assay); prior EFN report status of taxon	Histochemical/SEM survey will reveal active secretory structures in a significantly higher proportion of woody taxa with no prior visual EFN record than expected if true absence (rather than detection failure) explained the existing record	Paired histochemical/SEM survey of woody species with and without prior visual EFN reports, stratified by lineage (e.g., Salicaceae, Fabaceae)
H2—Ontogenetic decline in inducibility	Regulatory (Layer 1)	Tree/organ age and degree of secondary growth; amplitude and kinetics of nectar upregulation after herbivory or exogenous jasmonate	Induced nectar output (volume and/or secretion duration) will be negatively associated with tree/organ age, with mature trees showing lower-amplitude and/or slower-onset upregulation than juvenile trees of the same species following equivalent herbivory or jasmonate treatment	Common-garden or field cohort of *I. polycarpa* spanning juvenile to mature stages; standardized herbivory/jasmonate induction assay with nectar quantification
H3—Direct/indirect defense partitioning	Functional (Layer 1)	Relative defensive contribution of salicinoid-based direct defense vs. EFN-mediated ant defense, compared between specialist (salicinoid-adapted) and generalist herbivore guilds	EFN-mediated ant defense will confer a proportionally greater reduction in herbivory and/or mass gain for the salicinoid-adapted specialist (*Cerura vinula*) than for the generalist (*Lymantria dispar*), consistent with a guild-partitioned defense in which indirect defense compensates where direct chemical defense is least effective—rather than the two strategies conferring comparable, non-differential protection across both guilds	Factorial feeding assay with ant-exclusion, comparing specialist vs. generalist herbivore performance on *I. polycarpa*, paired with salicinoid quantification, following the design of Feistel et al. [77]
H4—Density-dependent associational resistance	Community (Layer 2)	Proportion of EFN-bearing trees in a stand; herbivory and ant visitation on neighboring non-EFN trees	Associational resistance (herbivory reduction on non-EFN neighbors) will increase non-linearly with the proportion of EFN-bearing trees in a stand, with a threshold proportion below which the effect is not statistically detectable	Plot-level manipulation of EFN-bearing tree proportion in replicated forest stands (extending the design of Staab et al. [72]), with herbivory and ant census on non-EFN neighbors

## Data Availability

No new data were created or analyzed in this study. Data sharing is not applicable to this article.
